# A stepwise framework for the normalization of array CGH data

**DOI:** 10.1186/1471-2105-6-274

**Published:** 2005-11-18

**Authors:** Mehrnoush Khojasteh, Wan L Lam, Rabab K Ward, Calum MacAulay

**Affiliations:** 1 British Columbia Cancer Research Centre, Vancouver, BC, Canada; 2 Department of Electrical and Computer Engineering, University of British Columbia, Vancouver, BC, Canada

## Abstract

**Background:**

In two-channel competitive genomic hybridization microarray experiments, the ratio of the two fluorescent signal intensities at each spot on the microarray is commonly used to infer the relative amounts of the test and reference sample DNA levels. This ratio may be influenced by systematic measurement effects from non-biological sources that can introduce biases in the estimated ratios. These biases should be removed before drawing conclusions about the relative levels of DNA. The performance of existing gene expression microarray normalization strategies has not been evaluated for removing systematic biases encountered in array-based comparative genomic hybridization (CGH), which aims to detect single copy gains and losses typically in samples with heterogeneous cell populations resulting in only slight shifts in signal ratios. The purpose of this work is to establish a framework for correcting the systematic sources of variation in high density CGH array images, while maintaining the true biological variations.

**Results:**

After an investigation of the systematic variations in the data from two array CGH platforms, SMRT (Sub Mega base Resolution Tiling) BAC arrays and cDNA arrays of Pollack et al., we have developed a stepwise normalization framework integrating novel and existing normalization methods in order to reduce intensity, spatial, plate and background biases. We used stringent measures to quantify the performance of this stepwise normalization using data derived from 5 sets of experiments representing self-self hybridizations, replicated experiments, detection of single copy changes, array CGH experiments which mimic cell population heterogeneity, and array CGH experiments simulating different levels of gene amplifications and deletions. Our results demonstrate that the three-step normalization procedure provides significant improvement in the sensitivity of detection of single copy changes compared to conventional single step normalization approaches in both SMRT BAC array and cDNA array platforms.

**Conclusion:**

The proposed stepwise normalization framework preserves the minute copy number changes while removing the observed systematic biases.

## Background

Microarray-based Comparative Genomic Hybridization (array CGH) is used to detect the aberrations in segmental copy numbers at chromosomal loci represented by DNA clones with known genomic locations [1]. CGH microarrays typically contain tens of thousands of spotted DNA sequences such as those derived from bacterial artificial chromosomes (BACs). Sample DNA from a test and a reference genome are labelled with different fluorescent dyes (usually Cyanine-3 and Cyanine-5 dyes) and then hybridized to the genomic microarray. The fluorescent signal intensity of each spot on the microarray serves as a relative measure of the amount of sample DNA bound to the DNA sequence of that spot. The ratio between the Cyanine-3 and the Cyanine-5 intensity of each spot reflects the relative quantities of the test and reference DNA samples.

The ratio of the two fluorescent signals at each spot is commonly used to detect copy number alteration. However, the ratios of the fluorescent signals are usually influenced by systematic effects from non-biological sources that can introduce biases in the estimates of these ratios. Such biases should be removed in order to draw conclusions on copy number status. The process of correcting for the systematic effects is often referred to as normalization.

Array CGH technology generally has more stringent performance requirements than gene expression microarray analysis. These requirements are to detect single DNA copy number changes in abnormal cells, typically within tumor samples. Detection sensitivity is complicated by the heterogeneous nature of tumor tissue with varying degrees of contamination from non-cancer cells. Due to the limitation in material availability performing replicate experiments is not always possible or desired.

In the context of developing a normalization protocol for array CGH, knowing the copy number status of DNA segments provides true values for calibration. The same copy number exists in different samples as the normal state for human cells is diploid. In contrast, gene expression level varies continuously for each gene and the expression level of the same gene is not expected to be identical in two different samples.

While a 2 fold change in signal may not represent a significant alteration in gene expression microarray analyses [3], for CGH arrays, a single copy gain compared to normal diploid DNA will result in a ratio of 3:2. A single copy loss would reduce the signal ratio to 1:2. Considering the contamination of tumor (abnormal) cells with non-cancer (normal) cells, the copy number ratio may be even smaller. So the challenge in normalization is to preserve the true copy number change signals while removing the systematic variations.

The purpose of this work is to correct for the systematic sources of variation while maintaining the true biological variations as small as a single copy number change in a sample of a heterogeneous cell population.

After an investigation of the systematic variations in the data from array CGH experiments, we tested existing normalization methods commonly used for gene expression data in order to deduce a stepwise normalization framework tailored to handling high density array CGH data. Here we demonstrate the efficacy of the stepwise normalization scheme through several quantitative characteristics of the data from several functional types of array CGH.

## Results and discussions

### Materials

Data from five sets of experiments were used in the development of the normalization strategy (Table 1). The first four datasets were generated from array CGH experiments performed using the SMRT (Sub Mega base Resolution Tiling) arrays. These arrays are tiling resolution BAC arrays with complete coverage of the human genome using 32,433 fingerprint-verified individually amplified BAC clones [4]. The experimental procedures for array CGH and generating spot images have been described previously [4]. The entire set of 32,433 solutions was spotted in triplicate onto two slides by a 4 × 12 pin arrayer. For the purpose of this study, only the data from the first array out of the two arrays were used.

**Table 1:** Data description. In this table, the array data of this study are summarized.

**Array**	**Reference DNA**	**Sample DNA**	**Data type for normalization performance evaluation**	**Evaluation method**
MM-1 to MM-4	Male genomic	Male genomic	Self-self hybridizations	S.d. for each array
H526-1 to H526-8	H526 cell line	Male genomic	Replicate H526 cell line experiments	1. Correlation coefficient for each pair of arrays 2. ICC 3. S.d. for each spot
MF-1 and MF-2	Female genomic	Male genomic	Single copy change	T-test
T1 to T5	Female genomic	Male/Female mixture (see Additional file 1)	Single copy loss with normal cell contamination	T-test
T6 to T10	Male genomic	Male/Female mixture (see Additional file 1)	Single copy gain with normal cell contamination	T-test
X1 to X5	Female DNA	cell lines containing varying numbers of X chromosomes (see Additional file 2)	varying levels of gene amplification and deletion for each of the X-chromosomal genes	T-test

The fifth dataset is a public dataset downloaded from the Stanford Microarray Database . This datasets was generated from array CGH experiments performed using human cDNA microarrays, [12].

The first dataset (self-self hybridization data) was derived from hybridization of the same DNA sample, i.e., normal male genomic DNA was used for both test and reference materials but labelled with different dyes. The four microarrays used in this CGH experiment are referred to as **MM-1 **to **MM-4 **in the following text.

The second dataset (hybridization data from replicate experiments) was derived from comparison of a tumor cell DNA sample with well characterized chromosomal aberrations (lung cancer cell line H526) [4] against normal male DNA. The 8 arrays used in this experiment are denoted **H526-1 **through **H526-8**.

The third dataset (hybridization data from male and female DNA mimicking single copy deletion) was derived from comparison of normal male DNA versus normal female DNA, using arrays named **MF-1 **and **MF-2**.

The fourth dataset (hybridization data from samples mimicking heterogeneous cell populations) was derived from a series of array CGH experiments in which the samples to be compared were mixtures of male and female DNA affecting X chromosome dosage mimicking tumor samples with varying levels of normal cell contamination. Precise proportions of DNA were mixed to simulate increasing levels of heterogeneity as previously described [5]. Arrays **T1 **through **T5 **compared male DNA against female DNA generating a 1:2 ratio for X chromosome sites mimicking a single copy deletion. Contamination from normal cells was then simulated by spiking varying amounts of female DNA into the male DNA sample. Arrays **T6 **through **T10 **compared a 50/50 mixture of male and female DNA against a male DNA reference generating a 3:2 ratio for X chromosome sites mimicking single copy amplifications. Contamination from normal cells was simulated by spiking varying amounts of female DNA into the male/female DNA mixture.

The fifth dataset was derived from hybridization of genomic DNAs from cell lines containing varying numbers of X chromosomes to simulate varying levels of gene amplification and deletion for each of the X-chromosomal genes present in the cDNA array [12]. The five experiments comprising the fifth data set are denoted **X1 **through **X5**.

### Systematic variations

After a thorough investigation of the systematic variations in the data from our array CGH experiments, four kinds of bias were identified. Below we explain each bias type.

#### Intensity bias

This bias is evident in the frequently used M-A plots which are plots of the log ratio *M *= log_2_(*I*_*r*_/*I*_*g*_) = log_2_(*I*_*r*_) - log_2_(*I*_*g*_) against the mean of the log intensities *A *= 1/2(log_2_(*I*_*r*_) + log_2_(*I*_*g*_)), where *I*_*r *_and *I*_*g *_are the intensities of the cyanine-5 and cyanine-3 channels respectively. In our data, this bias predominantly appears as curvature in the low intensity end of the M-A plot.

#### Spatial bias

**Figure 1:**
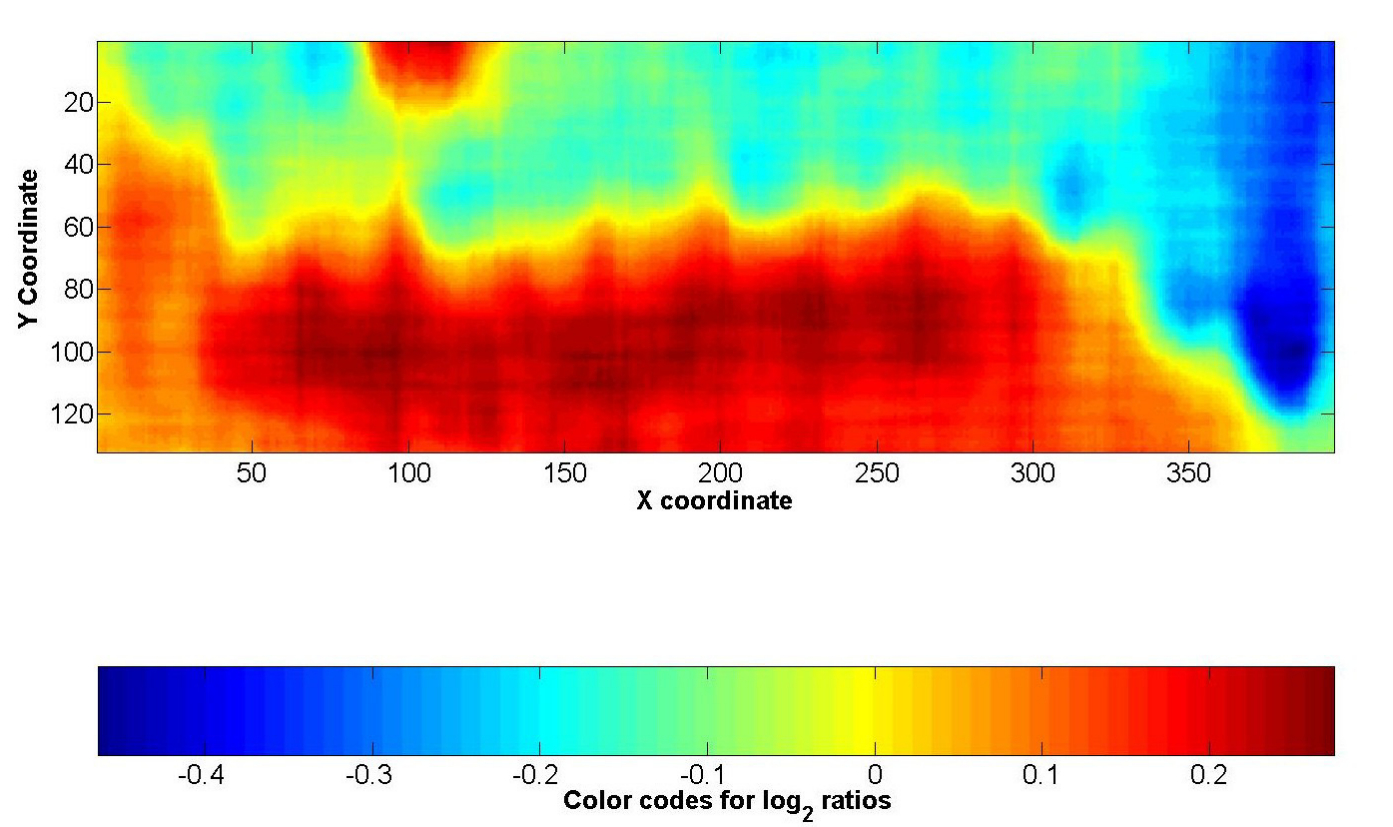
**A smoothed M-XY plot illustrating spatial bias**. The plot displays representation of log_2 _ratios based on the corresponding spot location on the microarray, the plot is smoothed with a moving median filter.

The representation of log ratios based on the corresponding spot location on the microarray is another type of plot which can be used to reveal spatially variable bias. We refer to this plot as M-XY plot. The spatially smoothed M-XY plot reveals the general trend of log ratios against their locations on the array (Fig. 1). For randomly distributed genomic loci across an array this plot should be a flat plane.

Spatial heterogeneity was thought to be caused by the different print tips used in printing the targets on the arrays [6]. However, our data show that the spatial heterogeneity is not caused by print tips effects because the spatial patterns are not organized in a block wise fashion (as they would be due to bias introduced by specific print tips). In fact, the patterns appear as a continuous function across the entire array.

#### Plate bias

This is a spatial pattern that can be seen in the data after the spatial gradient has been removed by the spatial normalization step mentioned above. This pattern is repeated in all subgrids in the M-XY plot and corresponds to the plate groups (groups of spots on the microarray that are all printed from the same microplate).

Plate bias is evident when box-plots of log_2 _ratios from each plate group are compared. These box plots show a systematic difference among the log_2 _ratios of the different plate groups. The median log_2 _ratio of each plate group is expected to be near zero, i.e. positive and negative deviations should cancel out in each plate group, unless the copy numbers of the clones in a plate biologically differ between the test and the control samples. We do not believe this is the case in our experiments.

This bias is caused by the fact that different clones that are produced in different microplates may have experienced slightly different physical conditions during the polymerase chain reaction (PCR) or in subsequent purification steps [7]. This variation in the efficiency of spot solution synthesis appears to affect different plate groups resulting in a plate level bias.

#### Background bias

The measured intensity for each microarray spot contains a contribution from the background fluorescence within the spot. This introduces a bias in the ratios of the spots' intensities. In the M-A plot this bias appears as deviation from zero in the log_2 _ratios of the lower intensity spots.

### Methods of bias removal

In order to remove these types of biases, we evaluated the following stepwise normalization procedure:

1. The spatial trend is estimated by computing, for each spot on the array, the median of log_2 _ratios for the spots within a spatial neighbourhood window of size 11 rows by 11 columns centred on that spot. The spatial bias that is estimated for each spot in this way is then subtracted from the log_2 _ratio of that spot. This step is referred to as "**Spatial**" normalization.

2. The plate bias is removed by calculating the median of the log_2 _ratios for all spots in the same plate group and subtracting it from the log_2 _ratios for all those spots. This step is referred to as "**Plate**" normalization.

3. The intensity bias is estimated using robust LOWESS curve fitting [8]. After this bias is estimated, assuming the bias is multiplicative; the bias is subtracted from the log ratios. This step is denoted as "**Intensity LOWESS**" normalization.

4. To remove the **background bias**, one of the following two different approaches is usually taken: either the estimate of the background intensity is subtracted from the estimated foreground intensity of each spot before taking the ratios, or it is not subtracted. In the latter case, the introduced bias is dealt with by treating it as intensity dependent bias. We evaluated both of these approaches in our experiments (see below).

Below we show that the above stepwise procedure is effective in removing the mentioned types of systematic variations. We demonstrate the efficacy of our procedure by comparing several quantitative characteristics of data normalized by our proposed strategy to those of non-normalized data and data normalized by other techniques listed in Table 2.

**Table 2:** Summary of normalization methods. Each of the normalization methods in this table will be denoted by its number through out the text. For full description of methods refer to "Methods of bias removal" section in Results and Discussion and the "Normalization methods" section in Methods.

**Method no.**	**Normalization method**	**Description**
**Background subtracted**		

**1**	No normalization	Raw ratios

**Global method**		

**2**	Global median Ratio	Ratios scaled by their median

**Intensity dependant methods**		

**3**	Intensity LOWESS, 10% span	Global Intensity LOWESS, span = 10%
**4**	Intensity LOWESS, 25% span	Global Intensity LOWESS, span = 25%
**5**	Intensity LOWESS, 40% span	Global Intensity LOWESS, span = 40%

**Spatial methods**		

**6**	Print tip mean Ratio	Ratios of each print-tip group scaled by the mean ratio of that group
**7**	Spatial	median of log2 ratios for the spots within a spatial neighbourhood window of size 11 rows by 11 columns centred on that spot
**8**	Spatial + Median Plate Ratio	Method 8 followed by plate normalization

**Combined intensity dependent and spatial methods**		

**9**	Print Tip Intensity LOWESS, span = 40%	LOWESS performed on the ratios from each print-tip group
**10**	Intensity LOWESS + Spatial	Stepwise Method 4 and 8

**Three step normalization**		

**11**	Intensity LOWESS + Spatial + Median Plate Ratio	Stepwise Methods 4 and 9
**12**	Spatial + Median Plate Ratio + intensity LOWESS	Stepwise Methods 9 and 4

**Background not subtracted**		

**13**	No Normalization	See Method 1, but without background subtraction

**Global method**		

**14**	Global median Ratio	See Method 2, but without background subtraction

**Intensity dependant methods**		

**15**	Global Intensity LOWESS, span = 10%	See Method 4, but without background subtraction

**Spatial methods**		

**16**	Print tip Mean Ratio	See Method 3, but without background subtraction
**17**	Spatial	See Method 8, but without background subtraction

**Combined intensity dependent and spatial methods**		

**18**	Intensity LOWESS + Spatial	See Method 10, but without background subtraction

**Three step normalization**		

**19**	Intensity LOWESS + Spatial + Median Plate Ratio	See Method 11, but without background subtraction

### Normalization of self-self array CGH data

The self-self experiments (arrays **MM-1 **through **MM-4**) were used to study the effect of normalization on removing the bias from the data and increasing the accuracy of the measurements. The 19 methods of normalization listed in Table 2 were evaluated on the data obtained from these arrays.

**Figure 2:**
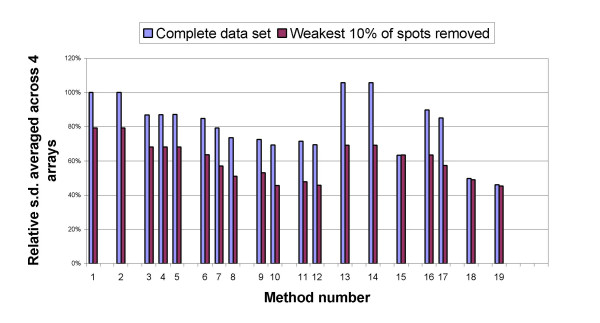
**Normalization of self-self hybridization data**. Relative standard deviation (s.d.) of log2 ratios averaged across arrays MM-1 through MM-4 using all data points are shown in blue. The repeated analysis of relative s.d. after removal of the weakest 10% of spots is shown in red. The numbers on the horizontal axis refer to the methods used for normalization listed on Table 2.

Since the same male genomic DNA serves as both sample and reference DNA, the copy numbers detected in both the Cyanine-3 and Cyanine-5 channels are expected to be the same at all loci, resulting in a zero theoretical value for the log_2 _ratio of intensities at all spots on the array. The effects of normalization on removing the bias were examined by calculating the standard deviation (s.d.) of the log_2 _ratios for each array in the experiment, evaluating each of the 19 methods listed in Table 2. Then all 19 standard deviations were scaled against the standard deviation of the raw ratios before normalization (i.e. against the s.d. value from the first method of Table 2). For each normalization method, the scaled s.d. values were then averaged across the four arrays. Figure 2 shows these average standard deviations.

The three different window sizes of 10%, 25% and 40% of the data points, used for LOWESS intensity normalization (methods 4-6 in Table 2) did not have a significant effect on the effectiveness of normalization.

Among 12 normalization methods that are performed on the ratios of background subtracted intensities, the stepwise strategy (method 12) results in the lowest s.d. for all four arrays. Also, among 7 normalization methods that are performed on the ratios of non-background subtracted intensities, the stepwise strategy (method 19) results in the smallest s.d.

When the three-step proposed normalization is performed on the ratios of non-background subtracted intensities, it yields better performance, in terms of reducing the s.d. of log_2 _ratios, than when it is applied to the ratios of background-subtracted intensities.

To further explore the effect of the background intensities, the standard deviations were recalculated for these four arrays with the lowest intensity spots removed from each data set. The difference between the s.d. of the ratios after normalization for the case of background subtracted and the case of non-background subtracted intensities became smaller on the reduced datasets. As an example, the new s.d. values when 10% of the lowest intensity spots are removed, are plotted in Fig. 2. This suggests that subtracting background increases the variability of ratios of lower intensity spots and the variability of higher intensity spots are not affected much by subtracting or not subtracting the background.

### Normalization of hybridization data from replicate experiments

In order to see how normalization affects the consistency of the data from replicate experiments, 8 replicate experiments were performed. **H526-1 **through **H526-8 **represent independent array CGH experiments using the same source of sample DNA (isolated from the well studied lung cancer cell line H526).

**Figure 3:**
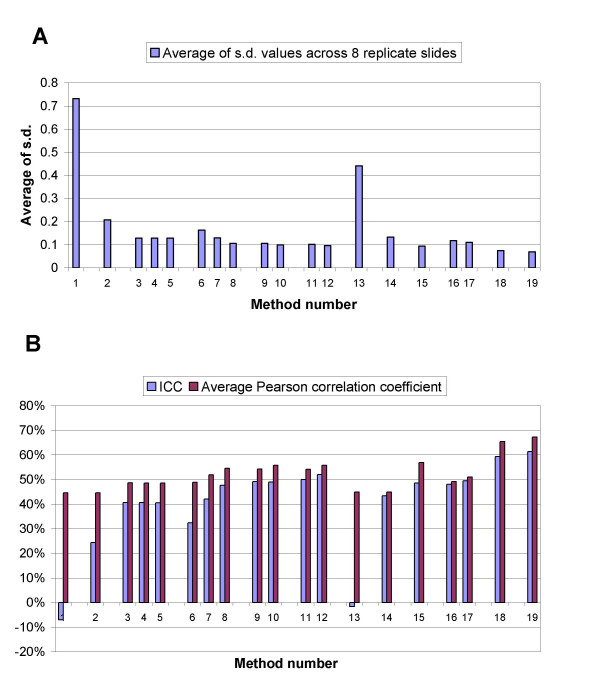
**Normalization of hybridization data from replicate experiments**. 8 replicate array CGH experiments were done comparing sample DNA from H526 cell line and the reference normal male genomic DNA. A. Graph shows the average of the standard deviations of log_2 _ratios for the same spot across 8 replicate arrays. B. shows the ICC and Average correlation coefficient of replicate arrays. Horizontal axis represents the method number listed in Table 2.

The *Standard deviations *of the log_2 _ratios of the same spot across the 8 replicate arrays were calculated and averaged across all the spots for each normalization method. The results are shown in Fig. 3A. The standard deviation measure attains its smallest value after method 12 or 19 is performed on the data. When the three-step normalization is performed on the ratios of non-background subtracted data (method 19), its performance is slightly better than when it is performed on ratios of background subtracted intensities (method 12).

The *Pearson's Correlation Coefficient *[9] was calculated for the data from each pair of the replicate arrays, with 28 possible pairings. The average of the 28 correlation coefficients for each single method was then calculated (Fig. 3B).

The *Intraclass Correlation Coefficient *(ICC) [9] was calculated for the set of data obtained from the 8 replicate arrays normalized using each of the methods described above. The results are also summarized in Fig. 3B. The ICC and Pearson correlation coefficient show similar results across the methods. Both ICC and Correlation coefficient attain their highest values after the three-step normalization method. This applies to both the ratios of non-background subtracted intensities and ratios of background subtracted intensities. ICC and Correlation coefficient are slightly higher when background subtraction is not performed on spot intensities measures.

### Normalization of hybridization data from male and female DNA

To evaluate the effect of normalization on improving detection of single copy loss, two array CGH experiments were conducted comparing male (XY) genomic DNA against female (XX) genomic DNA. The copy numbers of autosomal loci (clones on chromosome 1 through 22) are equal, while the X loci exhibit a 1:2 ratio, simulating a single copy loss.

**Figure 4:**
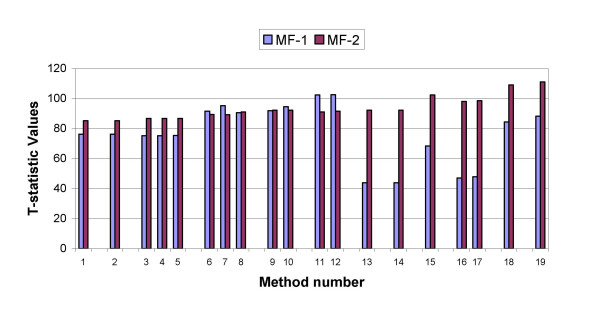
**Normalization of hybridization data from male and female DNA**. For each of arrays MF-1 and MF-2, a T-test was performed on the two groups of log ratios, i.e. log ratios for the autosomal clones and those for the X chromosome clones. Values of T-statistic after each normalization method are shown. Horizontal axis represents the method number listed in Table 2.

The normalization methods described above were applied to the data obtained from these two experiments. To determine which method results in the best separation of clones with normal copy from those with a single copy loss, a two-sample two-tailed T-test was performed on each array data normalized by each method. The T-test evaluates the difference between the means of two groups of log ratios. The first group consists of log ratios for clones from chromosomes 1 through 22 and the second group consists of log ratios for clones from chromosome X. The value of the T statistic is shown in Fig. 4 for both arrays and for each normalization method. A larger value for the T-statistic indicates better separation between the means of the two samples.

For the data from array **MF-1**, the largest T-statistic was obtained after our three-step normalization procedure was performed on the ratios of background subtracted intensities. For this array, the normalization methods performed on the ratios of the non-background subtracted intensities were not as effective.

For the **MF-2 **array data, the normalization methods do not significantly change the value of the T-statistic. The three-step normalization performed on the ratio of non-background subtracted intensities slightly increases the T-statistic. In fact the correlation coefficient of the log ratios and the estimated intensity bias and the correlation coefficient of the log ratios and the estimated spatial bias were both quite low for this array compared to the other arrays (below 15%). Also the background intensities for this array were quite low compared to the other arrays. This suggests that the reason for the lack of significant change in the T-statistic values after normalization is that the data from this particular array did not have significant bias.

### Normalization of hybridization data from samples mimicking heterogeneous cell populations and single copy alterations

Array CGH is often used to detect genetic alterations in tumor cells. However, tumours generally consist of heterogeneous cell populations including a variety of infiltrating non-cancerous cells. Contamination from normal cells may affect the ability to detect copy number aberrations. In the case of a single copy gain, contamination from diploid normal cells dampens the expected 3:2 signal ratio produced by the single copy gained sequences in the tumour cells due to the averaging effect in the mixed cell population. In the case of a single copy loss, normal cell contamination increases the average copy number, deviating from the expected 1:2 ratio. In a previous study, this effect on detection sensitivity was evaluated by mixing male (XY) and female (XX) DNA in precise proportions to mimic 0%, 15%, 30%, 50% and 75% normal cell contamination affecting the dosage of the X chromosome [5].

**Figure 5:**
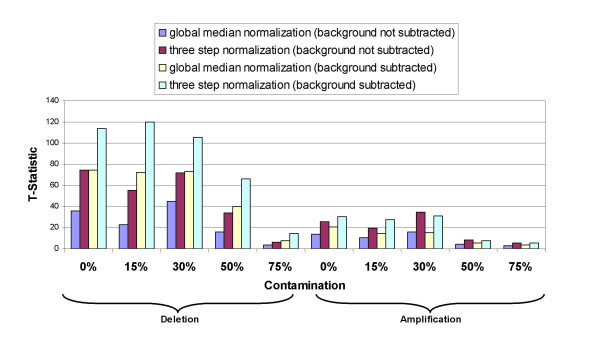
**Normalization of hybridization data from samples mimicking heterogeneous cell populations and single copy alterations**. Array CGH data were generated for samples mimicking single copy loss (deletion) or single copy gain (amplification) with contamination of increasing proportion of reference DNA, indicated as percentage on the horizontal axis. The experimental procedure for the array CGH experiments was previously described [5]. Global median normalization (method 1), stepwise normalization (method 12), global median normalization with background subtraction (method 13), and 3 step normalization with background subtraction (method 19) were applied. T-statistic values computed before and after normalization for arrays T1-T10 are summarized.

In this study, we wish to determine how our three-step normalization method affects the estimated log_2 _ratios for the clones with single copy number changes and increasing levels of heterogeneity. The stepwise normalization method was applied to the data from the titration series (arrays **T1**-**T10**) that simulated different contamination levels for both single copy gains and losses (Fig. 5).

We compared the data obtained after performing the three-step normalization procedure to data obtained after performing global median normalization on both the ratios of background subtracted intensities and the ratios of non-background subtracted intensities. For each array, a T-test was performed on the two groups of log ratios, i.e. log ratios for the autosomal clones and those for the X chromosome clones. T-values are shown in Fig. 5.

The T-statistic values are higher after normalization in all cases which assures us that the separation of the two groups is increased and the low-level copy number changes are preserved and even magnified. Comparing the T-statistic values for data with no normalization to the normalized results shows that normalization increases the sensitivity of detection of the single copy number changes up to 5 times. However, the T-statistic values are considerably lower for the ratios of non-background subtracted intensities as compared to the ratios of background subtracted intensities.

Functional normalization increases the separation between the distributions of the clones with normal and abnormal copy numbers and this facilitates the analysis of heterogeneous samples. For example, after normalization, the T-statistic for array **T9 **which simulates a single copy amplification with 50% contamination, becomes quite close to the T-statistic of array **T6 **which simulates a case with no contamination.

### Normalization of hybridization data from cDNA arrays simulating varying levels of gene amplification and deletion for X-chromosomal genes on the array

To evaluate the performance of the stepwise normalization strategy on hybridization data from cDNA arrays, we used public data from hybridization of genomic DNAs from cell lines containing varying numbers of X chromosomes that simulate varying levels of gene amplification and deletion for each of the X-chromosomal genes present on the array (arrays **X1 **to **X5**).

**Figure 6:**
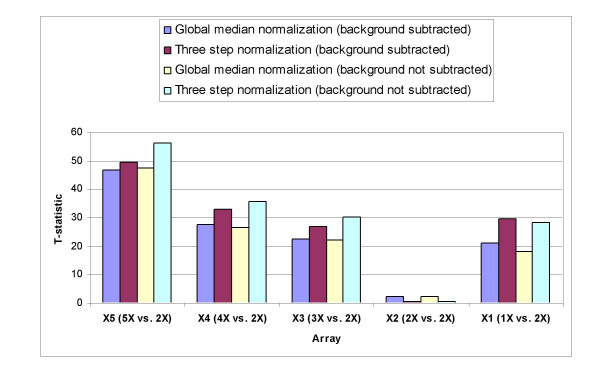
**Normalization of hybridization data from cDNA arrays**. Array CGH data were generated for samples simulating varying levels of gene amplification and deletion for X-chromosomal genes on the array. Global median normalization (method 1), stepwise normalization (method 12), global median normalization with background subtraction (method 13), and 3 step normalization with background subtraction (method 19) were applied. T-statistic values computed before and after normalization for arrays X1-X5 are summarized.

We compared the data obtained after performing the three-step normalization procedure to data obtained after performing global median normalization on both the ratios of background subtracted intensities and the ratios of non-background subtracted intensities. For each array, a T-test was performed on the two groups of log ratios, i.e. log ratios for the autosomal clones and those for the X chromosome clones. The T-statistic values are shown in Fig. 6.

The T-statistic values are higher after normalization in all cases. The increase in the T-statistic values may be interpreted as the increase in the separation of the distributions of the log_2 _ratios from two groups of normal and altered genes.

### Other considerations

#### Visual comparison of the genomic profiles

The use of the genomic location of the clones allows us to compare profiles before and after normalization and to use the visual correlation between observed and expected profiles as a measure of success. (This is not possible when analyzing gene expression array data.)

**Figure 7:**
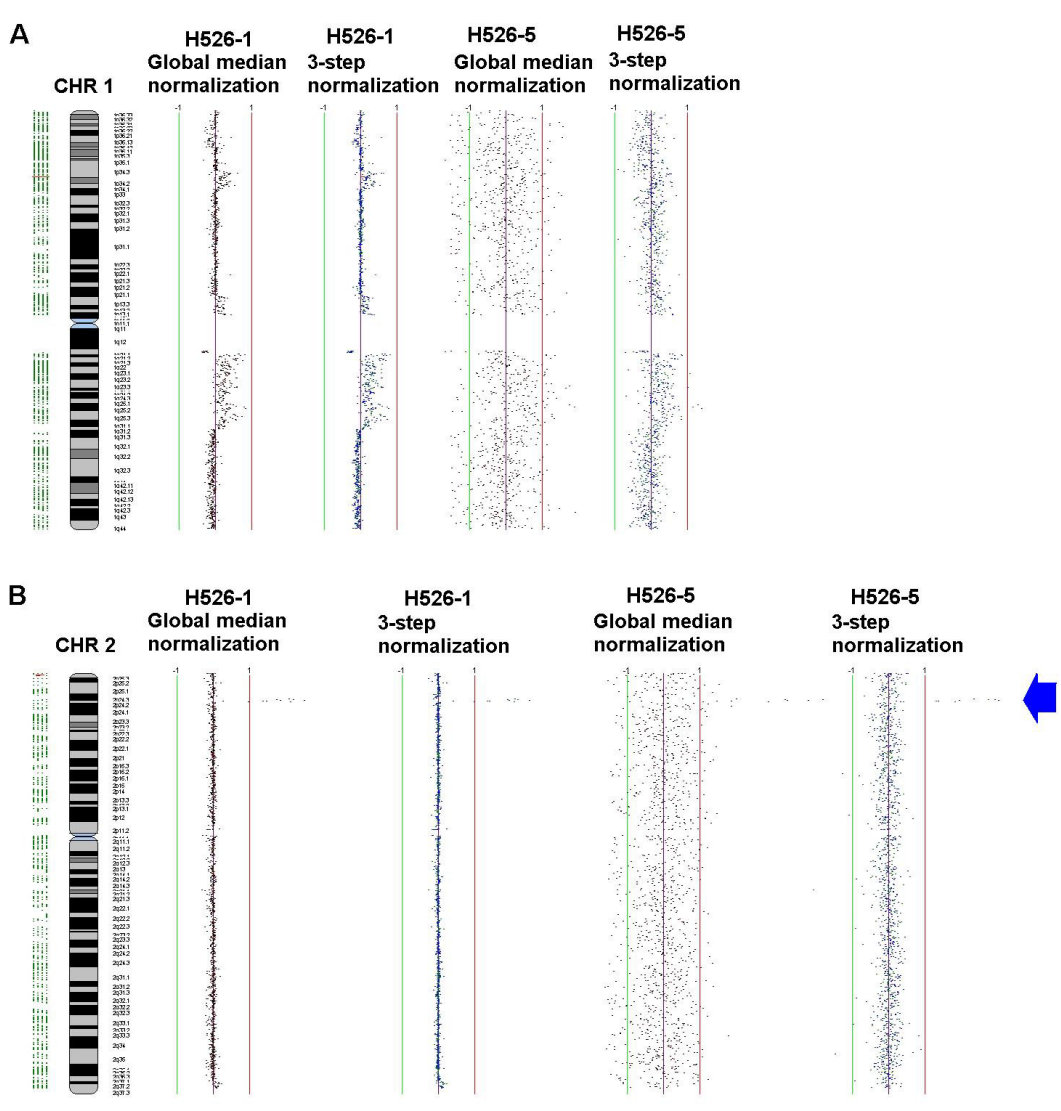
**Chromosome plots before and after normalization**. Plot of log_2 _signal ratios for clones (from chromosome 1 in A and chromosome 2 in B) versus their location across the chromosome. The profiles from left to right are: H526-1 data with global median normalization (method 1), H526-1 data with stepwise normalization (method 12), H526-5 data with global median normalization (method 13), H526-5 data with stepwise normalization (method 19). Each dot on the SeeGH plot represents a BAC clone. A shift in signal ratio to the left of center line indicates a copy number reduction, while a shift to the right indicates a gain. Blue arrow points to a high level segmental amplification. The arrow in part B points to the micro-amplification.

In Figures 6A and 6B, chromosome plots of the data from two of the replicate H526 arrays, generated by SeeGH software [10], are shown. Chromosome plots show the log_2 _of ratios for each of the target DNA clones, as a function of the location of the clone in the chromosome. Figure 7A shows the chromosome plots for chromosome 1 of arrays **H526-1 **and **H526-5**. Figure 7B shows the chromosome plots for chromosome 2 of arrays **H526-1 **and **H526-5**. For each array and each chromosome the log_2 _ratios are shown after global median normalization and after the three-step normalization. The variability of log_2 _ratios in array **H526-5 **is much higher than that of array **H526-1**. For the H526 genome, the regions of copy number changes are known [4]. As the figures show, for data from array **H526-5 **(low quality data), normalization reduced the unwanted variations. Consequently, after normalization the altered regions are clearer. An important point to note for data from array **H526-1 **(high quality data), where the variation of the log_2 _ratios is quite low even before normalization, is that normalization did not remove the true biological variation present in the sample.

#### Background subtraction

The issue of subtracting or not subtracting background intensities has been an open question in microarray data analysis. Some groups choose to use the raw intensities while others use the background subtracted intensities. Through our experiments we observed that not subtracting the background results in slightly less variability and more repeatability of the ratios. However, knowing the truth about the ratios of array CGH experiments enabled us to examine how subtracting and not subtracting the background intensities affect the ability to detect copy number changes. We observed that for the array CGH data from SMRT arrays the ability to detect the copy number changes when using the ratio of non-background subtracted intensities is degraded when compared to using the ratio of background subtracted intensities. However, for the array CGH data from cDNA arrays, the ability to detect the copy number changes when using the ratio of non-background subtracted intensities is increased. We believe that the fact that different methods of background estimation are used in these two cases and the differences in the average level of background intensities of the arrays have caused this inconsistency between the results. The data from SMRT arrays along with the image analysis methods used suggest that background subtraction improves normalization and should be performed for these data.

## Conclusion

We evaluated the performance and effectiveness of an integration of novel and existing bias removal methods mainly used for gene expression arrays considering the stringent performance requirements of the array CGH experiments and using the characteristics of the array CGH data that provide the true biological values for calibration.

A normalization scheme is expected to remove the systematic variations in the data and leave the true biological variations unchanged. In evaluating the performance of the normalization methods, both these issues should be considered. Our method is shown to preserve even the low-level copy number changes while reducing the systematic biases. To the best of our knowledge this is the first study to examine the effectiveness of various normalization methods taking advantage of the knowledge of the underlying truth in known copy number status in genomic array CGH data – as opposed to using variable gene expression changes in normalizing expression microarray data. Our stepwise normalization framework estimates the intensity dependent, spatial and plate bias using regression-based techniques and removes the estimated biases from the raw log_2 _ratios. These biases were observed in two different array CGH platforms, the SMRT BAC arrays [4] and cDNA arrays [12]. Our results demonstrate that multi-step normalization outperforms conventional single step methods in reducing systematic biases in array CGH spot data from both BAC and cDNA platforms (such as those representing self-self hybridization, replicated experiments, single copy detection, and data mimicking tissue heterogeneity) and suggest that multiple systematic variations need to be addressed in the normalization of genomic array CGH data.

In this study we focused on within-array normalization and did not consider performing between-array normalization. This was based on the fact that because of tissue heterogeneity, there is usually some degree of contamination from normal cells into the tumor cells in array CGH experiment samples. As a result, it is not known that a single copy change results in how much change in the fluorescent ratios [5]. Because of this, it seems that the safest way to deal with the issue of unequal scales of data from different arrays would be to find the regions of gains or losses in DNA copy number according to data from one array CGH experiment and assign different levels of change to those different regions. These levels may then be compared across arrays.

## Methods

### Microarray image analysis

#### SMRT arrays

Hybridized arrays were imaged using a charge-coupled device based imaging system and analyzed using the SoftWorx Tracker spot analysis software (ArrayWorx eAuto, API, Issaquah, WA). The mean pixel intensity was used for the spot foreground intensities and the median pixel intensity was used for the spot background intensity. Background calculation was achieved using the "Cell method" in the SoftWorx Tracker program. In this method, a square of 125% size of spot spacing is drawn and centred on the centroid of the spot's contour. All pixels within the square which are not located within the two-pixel margin of the spot's contour are treated as background pixels for that spot.

#### cDNA arrays

The image analysis methods are described in [12]. For computing the fluorescence ratios, the mean pixel intensity was used for the spot foreground intensities and the median pixel intensity was used for the spot background intensity.

### Normalization methods

The normalization methods that were used in this study are listed in Table 2. Among these methods, global intensity LOWESS, Median Plate Ratio and Spatial normalization have been described in the text above. For comparison purposes print tip LOWESS intensity normalization [6] is also implemented, performing LOWESS curve fitting on log ratios from each subgrid of the microarray.

LOWESS (Locally Weighted Scatter plot Smoothing) is a curve-fitting technique based on local regression [8]. Each smoothed value is determined by its neighbouring data points defined within the span. A regression weight function is defined for the data points contained within the span. In addition to the regression weight function, a robust weight function may be used, which makes the process resistant to outliers. In this study, we used a robust LOWESS with a first degree polynomial for regression.

### Evaluation methods

#### Pearson's correlation coefficient

In the analysis of replicated experiments (H526-1 through H526-8), the Pearson correlation coefficient is calculated for all 28 possible pairings of the 8 replicate arrays. For each pair wise comparison, the log_2 _ratios for spots from one array form the first group, and the log_2 _ratios for the corresponding spots from the other array form the second group.

#### Intra-class correlation coefficient (ICC)

This is an ANOVA-based type of correlation. It measures the relative homogeneity within groups compared to their total variation. Suppose that we have *k *groups of measurements and each group consists of *n *replicate measurements. X_i,j_, i = 1,..,k and j = 1,..,n represents the j-th measurement in the i-th group. If we define:

X¯=∑i=1k∑j=1nXi,jnk
 MathType@MTEF@5@5@+=feaafiart1ev1aaatCvAUfKttLearuWrP9MDH5MBPbIqV92AaeXatLxBI9gBaebbnrfifHhDYfgasaacH8akY=wiFfYdH8Gipec8Eeeu0xXdbba9frFj0=OqFfea0dXdd9vqai=hGuQ8kuc9pgc9s8qqaq=dirpe0xb9q8qiLsFr0=vr0=vr0dc8meaabaqaciGacaGaaeqabaqabeGadaaakeaacuWGybawgaqeaiabg2da9maalaaabaWaaabCaeaadaaeWbqaaiabdIfaynaaBaaaleaacqWGPbqAcqGGSaalcqWGQbGAaeqaaaqaaiabdQgaQjabg2da9iabigdaXaqaaiabd6gaUbqdcqGHris5aaWcbaGaemyAaKMaeyypa0JaeGymaedabaGaem4AaSganiabggHiLdaakeaacqWGUbGBcqWGRbWAaaaaaa@44C1@

X¯i=∑j=1nXi,jn
 MathType@MTEF@5@5@+=feaafiart1ev1aaatCvAUfKttLearuWrP9MDH5MBPbIqV92AaeXatLxBI9gBaebbnrfifHhDYfgasaacH8akY=wiFfYdH8Gipec8Eeeu0xXdbba9frFj0=OqFfea0dXdd9vqai=hGuQ8kuc9pgc9s8qqaq=dirpe0xb9q8qiLsFr0=vr0=vr0dc8meaabaqaciGacaGaaeqabaqabeGadaaakeaacuWGybawgaqeamaaBaaaleaacqWGPbqAaeqaaOGaeyypa0ZaaSaaaeaadaaeWbqaaiabdIfaynaaBaaaleaacqWGPbqAcqGGSaalcqWGQbGAaeqaaaqaaiabdQgaQjabg2da9iabigdaXaqaaiabd6gaUbqdcqGHris5aaGcbaGaemOBa4gaaaaa@3E01@

RSS=∑i=1k(X¯i−X¯)2
 MathType@MTEF@5@5@+=feaafiart1ev1aaatCvAUfKttLearuWrP9MDH5MBPbIqV92AaeXatLxBI9gBaebbnrfifHhDYfgasaacH8akY=wiFfYdH8Gipec8Eeeu0xXdbba9frFj0=OqFfea0dXdd9vqai=hGuQ8kuc9pgc9s8qqaq=dirpe0xb9q8qiLsFr0=vr0=vr0dc8meaabaqaciGacaGaaeqabaqabeGadaaakeaacqWGsbGucqWGtbWucqWGtbWucqGH9aqpdaaeWbqaamaabmaabaGafmiwaGLbaebadaWgaaWcbaGaemyAaKgabeaakiabgkHiTiqbdIfayzaaraaacaGLOaGaayzkaaWaaWbaaSqabeaacqaIYaGmaaaabaGaemyAaKMaeyypa0JaeGymaedabaGaem4AaSganiabggHiLdaaaa@3FEE@

TSS=∑i=1k∑j=1n(Xi,j−X¯)2
 MathType@MTEF@5@5@+=feaafiart1ev1aaatCvAUfKttLearuWrP9MDH5MBPbIqV92AaeXatLxBI9gBaebbnrfifHhDYfgasaacH8akY=wiFfYdH8Gipec8Eeeu0xXdbba9frFj0=OqFfea0dXdd9vqai=hGuQ8kuc9pgc9s8qqaq=dirpe0xb9q8qiLsFr0=vr0=vr0dc8meaabaqaciGacaGaaeqabaqabeGadaaakeaacqWGubavcqWGtbWucqWGtbWucqGH9aqpdaaeWbqaamaaqahabaWaaeWaaeaacqWGybawdaWgaaWcbaGaemyAaKMaeiilaWIaemOAaOgabeaakiabgkHiTiqbdIfayzaaraaacaGLOaGaayzkaaWaaWbaaSqabeaacqaIYaGmaaaabaGaemOAaOMaeyypa0JaeGymaedabaGaemOBa4ganiabggHiLdaaleaacqWGPbqAcqGH9aqpcqaIXaqmaeaacqWGRbWAa0GaeyyeIuoaaaa@4911@

*SSE *= *TSS *- *RSS*

MSbetweengroups=RSSk
 MathType@MTEF@5@5@+=feaafiart1ev1aaatCvAUfKttLearuWrP9MDH5MBPbIqV92AaeXatLxBI9gBaebbnrfifHhDYfgasaacH8akY=wiFfYdH8Gipec8Eeeu0xXdbba9frFj0=OqFfea0dXdd9vqai=hGuQ8kuc9pgc9s8qqaq=dirpe0xb9q8qiLsFr0=vr0=vr0dc8meaabaqaciGacaGaaeqabaqabeGadaaakeaacqWGnbqtcqWGtbWudaWgaaWcbaGaemOyaiMaemyzauMaemiDaqNaem4DaCNaemyzauMaemyzauMaemOBa4wbaeqabeqaaaqaaiabdEgaNjabdkhaYjabd+gaVjabdwha1jabdchaWjabdohaZbaaaeqaaOGaeyypa0ZaaSaaaeaacqWGsbGucqWGtbWucqWGtbWuaeaacqWGRbWAaaaaaa@474A@

MSwithingroups=SSEnk−k
 MathType@MTEF@5@5@+=feaafiart1ev1aaatCvAUfKttLearuWrP9MDH5MBPbIqV92AaeXatLxBI9gBaebbnrfifHhDYfgasaacH8akY=wiFfYdH8Gipec8Eeeu0xXdbba9frFj0=OqFfea0dXdd9vqai=hGuQ8kuc9pgc9s8qqaq=dirpe0xb9q8qiLsFr0=vr0=vr0dc8meaabaqaciGacaGaaeqabaqabeGadaaakeaacqWGnbqtcqWGtbWudaWgaaWcbaGaem4DaCNaemyAaKMaemiDaqNaemiAaGMaemyAaKMaemOBa4wbaeqabeqaaaqaaaaacqWGNbWzcqWGYbGCcqWGVbWBcqWG1bqDcqWGWbaCcqWGZbWCaeqaaOGaeyypa0ZaaSaaaeaacqWGtbWucqWGtbWucqWGfbqraeaacqWGUbGBcqWGRbWAcqGHsislcqWGRbWAaaaaaa@49AA@

then r_ICC _is calculated from the following formula:

rICC=MSBetweengroups−MSWithingroupsMSBetweengroups+(n−1)∗MSWithingroups
 MathType@MTEF@5@5@+=feaafiart1ev1aaatCvAUfKttLearuWrP9MDH5MBPbIqV92AaeXatLxBI9gBaebbnrfifHhDYfgasaacH8akY=wiFfYdH8Gipec8Eeeu0xXdbba9frFj0=OqFfea0dXdd9vqai=hGuQ8kuc9pgc9s8qqaq=dirpe0xb9q8qiLsFr0=vr0=vr0dc8meaabaqaciGacaGaaeqabaqabeGadaaakeaacqWGYbGCdaWgaaWcbaGaemysaKKaem4qamKaem4qameabeaakiabg2da9maalaaabaGaemyta0Kaem4uam1aaSbaaSqaaiabdkeacjabdwgaLjabdsha0jabdEha3jabdwgaLjabdwgaLjabd6gaUvaabeqabiaaaeaaaeaacqWGNbWzcqWGYbGCcqWGVbWBcqWG1bqDcqWGWbaCcqWGZbWCaaaabeaakiabgkHiTiabd2eanjabdofatnaaBaaaleaacqWGxbWvcqWGPbqAcqWG0baDcqWGObaAcqWGPbqAcqWGUbGBfaqabeqacaaabaaabaGaem4zaCMaemOCaiNaem4Ba8MaemyDauNaemiCaaNaem4CamhaaaqabaaakeaacqWGnbqtcqWGtbWudaWgaaWcbaGaemOqaiKaemyzauMaemiDaqNaem4DaCNaemyzauMaemyzauMaemOBa4wbaeqabeGaaaqaaaqaaiabdEgaNjabdkhaYjabd+gaVjabdwha1jabdchaWjabdohaZbaaaeqaaOGaey4kaSYaaeWaaeaacqWGUbGBcqGHsislcqaIXaqmaiaawIcacaGLPaaacqGHxiIkcqWGnbqtcqWGtbWudaWgaaWcbaGaem4vaCLaemyAaKMaemiDaqNaemiAaGMaemyAaKMaemOBa4wbaeqabeGaaaqaaaqaaiabdEgaNjabdkhaYjabd+gaVjabdwha1jabdchaWjabdohaZbaaaeqaaaaaaaa@8929@

The maximum positive value of the intra-class correlation coefficient is 1.0, but its maximum negative value is (-1/(n-1)). Intra-class correlation coefficient is large and positive when there is no variation within the groups, but the group means differ. Intra-class correlation coefficient is large and negative when the group means are the same but there is great variation within groups. A negative intra-class correlation occurs when between-group variation is less than within-group variation [9]. ICC was shown to be useful for the assessment of technical and biological variations in microarray experiments [11].

In evaluating the normalized data from the replicate arrays (H526-1 through H526-8), n is the total number of replicate arrays which is 8 and k is the total number of clones on each array, and X_i,j _represents the estimated log_2 _ratio for the j-th clone on the i-th array.

#### T-test

A two-sample two-tailed T-test was used to determine whether two samples (with different numbers of observations) from a normal distribution (in x and y) could have the same mean when the standard deviations are unknown but assumed equal.

In the analysis performed on third, fourth, and fifth datasets (**MF-1 **to **MF-2**, **T1 **to **T10**, and **X1 **to **X5**), for each array dataset, log_2 _ratios for autosomal clones represent the first sample and log_2 _ratios for clones from chromosome X represent the second sample.

## Authors' contributions

MK developed and implemented the methods, participated in the design of the study, and drafted the manuscript. WL provided expertise on the array CGH platform. RW participated in the coordination of the study. CM participated in the design, development and coordination of the study. All authors read and approved the manuscript.

## Supplementary Material

Additional File 1 **Supplemental table 1**. A description of array CGH experiments that simulate varying degrees of normal diploid cells contamination in a population of cancer cells carrying a single copy alteration. A more detailed description can be found in [5].
Additional file 2 - Supplemental table 2 Click here for file

Additional File 2 **Supplemental table 2**. A Description of array CGH experiments involving hybridization of genomic DNAs from cell lines containing varying numbers of X chromosomes that simulate varying levels of gene amplification and deletion for each of the X-chromosomal genes present on the cDNA array. A more detailed description can be found in .
Additional file 2 - Supplemental table 2 Click here for file
