# Supplementary material for: A stepwise framework for the normalization of array CGH data
Source: BMC Bioinformatics. 2005 Nov 18;6:274. doi: 10.1186/1471-2105-6-274 (PMC1310623; doi:10.1186/1471-2105-6-274)
Supplement: Additional File 1 — Supplemental table 1. A description of array CGH experiments that simulate varying degrees of normal diploid cells contamination in a population of cancer cells carrying a single copy alteration. A more detailed description can be found in [5]. Additional file 2 - Supplemental table 2 [file 1471-2105-6-274-S1.doc]

## Supplemental Table 1 - Simulation of tissue heterogeneity.

A description of array CGH experiments that simulate varying degrees of normal diploid cells contamination in a population of cancer cells carrying a single copy alteration. A more detailed description can be found in [5].

| **Array** | | **Sample DNA (mixture of**  **Male DNA and Female DNA)** | | **Reference DNA** | **X dosage in probe vs. reference** |
| --- | --- | --- | --- | --- | --- |
|  | | Male DNA | Female DNA |  | |
| Series for simulating single copy loss | T1 | 100% | 0% | Female | 1:2 |
|  | T2 | 85% | 15% | Female | 1.15:2 |
|  | T3 | 70% | 30% | Female | 1.3:2 |
|  | T4 | 50% | 50% | Female | 1.5:2 |
|  | T5 | 25% | 75% | Female | 1.75:2 |
| Series for simulating single copy gain | T6 | 50%% | 50% | Male | 1.5:1 |
|  | T7 | 57.5% | 42.5% | Male | 1.43:1 |
|  | T8 | 65% | 35% | Male | 1.35:1 |
|  | T9 | 75% | 25 | Male | 1.25:1 |
|  | T10 | 87.5% | 12.5 | Male | 1.13:1 |
