# Supplementary material for: A stepwise framework for the normalization of array CGH data
Source: BMC Bioinformatics. 2005 Nov 18;6:274. doi: 10.1186/1471-2105-6-274 (PMC1310623; doi:10.1186/1471-2105-6-274)
Supplement: Additional File 2 — Supplemental table 2. A Description of array CGH experiments involving hybridization of genomic DNAs from cell lines containing varying numbers of X chromosomes that simulate varying levels of gene amplification and deletion for each of the X-chromosomal genes present on the cDNA array. A more detailed description can be found in . Additional file 2 - Supplemental table 2 [file 1471-2105-6-274-S2.doc]

## Supplemental Table 2 - Simulation of **varying levels of gene amplification and deletion for X-chromosomal genes**

A Description of array CGH experiments involving hybridization of genomic DNAs from cell lines containing varying numbers of X chromosomes that simulate varying levels of gene amplification and deletion for each of the X-chromosomal genes present on the cDNA array. A more detailed description can be found in [http://smd.stanford.edu]

| Array | Sample | Reference | Slide name (as in Stanford microarray database) |
| --- | --- | --- | --- |
| X1 | 45, XO | 46, XX | svi112 |
| X2 | 46, XX | 46, XX | svi111 |
| X3 | 47, XXX | 46, XX | svi110 |
| X4 | 48, XXXX | 46, XX | svi109 |
| X5 | 49, XXXXX | 46, XX | svi108 |
